# MicroRNA Expression in Alpha and Beta Cells of Human Pancreatic Islets

**DOI:** 10.1371/journal.pone.0055064

**Published:** 2013-01-29

**Authors:** Dagmar Klein, Ryosuke Misawa, Valia Bravo-Egana, Nancy Vargas, Samuel Rosero, Julieta Piroso, Hirohito Ichii, Oliver Umland, Jiang Zhijie, Nicholas Tsinoremas, Camillo Ricordi, Luca Inverardi, Juan Domínguez-Bendala, Ricardo L. Pastori

**Affiliations:** 1 Diabetes Research Institute, University of Miami Leonard M. Miller School of Medicine, Miami, Florida, United States of America; 2 Center for Computational Science, University of Miami, Miami, Florida, United States of America; 3 Department of Medicine, University of Miami Leonard M. Miller School of Medicine, Miami, Florida, United States of America; 4 Department of Surgical Sciences, University of Miami Leonard M. Miller School of Medicine, Miami, Florida, United States of America; University of Bremen, Germany

## Abstract

microRNAs (miRNAs) play an important role in pancreatic development and adult β-cell physiology. Our hypothesis is based on the assumption that each islet cell type has a specific pattern of miRNA expression. We sought to determine the profile of miRNA expression in α-and β-cells, the main components of pancreatic islets, because this analysis may lead to a better understanding of islet gene regulatory pathways. Highly enriched (>98%) subsets of human α-and β-cells were obtained by flow cytometric sorting after intracellular staining with c-peptide and glucagon antibody. The method of sorting based on intracellular staining is possible because miRNAs are stable after fixation. MiRNA expression levels were determined by quantitative high throughput PCR-based miRNA array platform screening. Most of the miRNAs were preferentially expressed in β-cells. From the total of 667 miRNAs screened, the Significant Analysis of Microarray identified 141 miRNAs, of which only 7 were expressed more in α-cells (α-miRNAs) and 134 were expressed more in β-cells (β-miRNAs). Bioinformatic analysis identified potential targets of β-miRNAs analyzing the Beta Cell Gene Atlas, described in the T1Dbase, the web platform, supporting the type 1 diabetes (T1D) community. cMaf, a transcription factor regulating glucagon expression expressed selectively in α-cells (TFα) is targeted by β-miRNAs; miR-200c, miR-125b and miR-182. Min6 cells treated with inhibitors of these miRNAs show an increased expression of cMaf RNA. Conversely, over expression of miR-200c, miR-125b or miR-182 in the mouse alpha cell line αTC6 decreases the level of cMAF mRNA and protein. MiR-200c also inhibits the expression of Zfpm2, a TFα that inhibits the PI3K signaling pathway, at both RNA and protein levels.

In conclusion, we identified miRNAs differentially expressed in pancreatic α- and β-cells and their potential transcription factor targets that could add new insights into different aspects of islet biology and pathophysiology.

## Introduction

MicroRNAs (miRNAs) are small non-coding RNAs that negatively regulate gene expression by interacting with the 3′UTR of target mRNAs [1], [2]. miRNAs play a fundamental role in regulating gene expression in key biological events such as cell proliferation, differentiation, death and malignant transformation [2]. It has been shown that miRNAs regulate embryonic and organ development, including pancreatic specification and islet function [3], [4], [5], [6], [7], [8], [9], [10], [11], [12]. We have previously identified a subset of miRNAs differentially expressed in adult and developing human islets [13], [14]. However, studies of miRNA expression patterns in endocrine islet cell subsets have not yet been reported. We hypothesize that each cell type within the islet will have a specific miRNA expression pattern. A key requirement for this kind of study is the availability of efficient methods to obtain highly pure human α- and β-cell populations. Methods describing successful isolation of β-cells for mouse and rat pancreatic endocrine cells have been published [15], [16], [17], [18]. Dissecting β-cells directly from the pancreatic tissue using the laser capture microdissection (LCM) technique allowed the procurement of pure human and mouse β-cells [19], [20]. Human β-cells were identified by their intrinsic autofluorescence [20]. Recently, two publications described the isolation of human pancreatic endocrine cells by flow cytometric cell sorting (FACS). In one study the authors reported the isolation of functional human α-cells by improving the gating selection [21]; in the other, cell type-specific surface-reactive antibodies were used to label and separate all major cells of the human pancreas [22]. The isolation of human α-cells using the same criteria is more challenging because of the weak specificity of intrinsic autofluorescence. Furthermore, because in rodents the α-cells are situated on the periphery of the islet, distinctly separated from the β-cells, they can be collected by simple exclusion of the β-cell core. This method is more challenging for human islets because both cell populations are intertwined [23], [24].

Another alternative that has yielded highly purified mouse islet cells is the labeling of endocrine islet cells by targeting specific hormones such as insulin and glucagon [25]. This method requires fixation and permeabilization. Conventional fixation degrades mRNA and modifies it by cross linking with methylol groups, which makes the characterization of gene expression patterns in different cell populations more difficult [26]. Because of this limitation, alternative methods such as detection of mRNA in cell homogenates avoiding RNA isolation have been successfully utilized [25], [27]. However, miRNAs are resistant to fixation and can be recovered from fixed and permeabilized tissue [28], [29]. Taking advantage of this unique feature, we are able to study miRNA expression in highly purified populations of human α- and β-cells.

## Materials and Methods

### Isolation of islets

Human islets were obtained from the Diabetes Research Institute cell-processing facility or from other participating centers of the National Institute of Health Islet Cell Resources (ICR). The organs were obtained from deceased healthy multiorgan donors (between the ages of 28 and 54) who consented to research use. Islets of Langerhans were isolated from the pancreas using the automated method [30]. The protocols were reviewed and approved by the University Of Miami Institutional Review Board (IRB).

### Isolation of human α-and β-cells

Dissociated human islets were stained with 7-aminoactinomycin D (7AAD) to identify dead cells. The cells were fixed with 2.5% paraformaldehyde (10 min on ice) and permeabilized with a saponin-based buffer (BD Perm/Wash Buffer). The staining was performed with mouse anti human c-peptide monoclonal antibody (1∶100 Abcam Cambridge MA) or glucagon (1∶500 Dako Carpinteria CA). The secondary antibodies were APC-labeled goat anti mouse IgG (1∶200 Invitrogen, Molecular probes CA). C-peptide+/7AAD− and glucagon+/7AAD− populations were isolated by flow cytometric cell sorting (FACS). On average the sorted populations were >98% pure with the final yield ranging between 60 and 80%.

### MiRNA PCR array: quantitative miRNA profiling

Total RNA was isolated from sorted α-cells and β-cells, purified from 6 human pancreases, using the mirVana miRNA Isolation kit (Ambion/Invitrogen CA) (**Table S1**). cDNA synthesis and PCR amplification were performed according to manufacturer's instructions (Invitrogen-Applied Biosystems, Foster City, CA). MiRNA profiling was performed on the AB7900 instrument (Applied Biosystems) using micro fluidic cards TaqMan® Low Density Array (TLDA, v2.0) for human miRNAs, which allows quantitative assessment of 667 miRNAs. The cDNA from each sample was preamplified 12 cycles according to manufacturer's instructions and processed on cards A and B; the cut-off value was set at 32 cycles [31].

Fold changes were calculated with the Applied Biosystems SDS software, utilizing the equation RQ = 2^−ΔΔCt^, where Ct is the threshold cycle to detect fluorescence for a particular miRNA [32]. ΔΔCt = [(Ct_β_−Ct_Ctrl-RNAβ_)−(Ct_α_−Ct_Ctrl-RNAα_)]. The endogenous control -Ctrl-RNA- is RNU48, a control small nucleolar RNA. The data have been deposited in the database Gene Expression Omnibus (GEO) (GSE38360). For RQ calculations samples with CTs>32 were considered as 32.

### Quantitative RT-PCR (qRT-PCR)

Total RNA was isolated using the mirVana miRNA Isolation kit (Ambion). cDNA synthesis and PCR amplifications were done with TaqMan reagents following manufacturer's protocol (Applied Biosystems). Quantification was carried out with the 7500 Fast Real-time PCR system (Applied Biosystems). RNA expression was calculated as Relative quantification (RQ). RQ = 2^−ΔCt^, ΔCt = (Ct_RNA gene_−Ct_RNA Ctrl_), Ct≥35 cycles was considered as undetermined. The number of amplification cycles (Cts) was normalized to the endogenous control beta-actin. The same RNA isolation method was used to assess miRNAs in alphaTC6 and Min6 cells using Taqman primers and U6 small nuclear RNA as endogenous control.

### Luciferase Assay

The glucagon expressing αTC6 cells (ATCC) were grown in DMEM medium containing 2 mg/ml glucose and 10%FBS. The cells were seated in 6 well plates 24 hours before transfection at 50% confluency. To asses if cMAf is a target for miR-200c, the firefly Luciferase was used as a reporter gene. Normalization was done using Renilla Luciferase. Both activities were measured in succession in the same sample. The pEZX- MT01-cMaf 3′UTR-fLuc (GeneCopoeia; Rockville MD) vector (1 µg) was transfected into cells with Dharmafect duo (Dharmacon -Thermo Scientific Lafyate CO) alone or together with either mimic miR-200c (50 nM) or irrelevant mimic (50 nM) (Thermo Scientific –Dharmacon). Transfected cells were cultured 18–20 hrs at 37°C plus 5%CO2 and then transferred to opaque flat bottom white 96 well plates (BD Biosciences San Jose CA) and cultured under the same conditions for additional 24 hrs. The evaluation of the experiment was performed using Dual Luminescence assay kit (GeneCopoeia MD). The culture medium was removed and replaced by 100 µl of lyses buffer containing firefly luciferase substrate and allowed to incubate for 10 minutes before reading. Immediately after the first reading 100 µl of firefly luciferase quenching buffer containing substrate for Renilla was added and after 10 minutes incubation the normalizing fluorescence was acquired. Both readings were performed on Spectra Max M5 plate reader (Molecular Devices; Sunnyvale CA).

### Overexpression of miRNAs in α-TC6 cells

The α-TC6 (ATCC) cells were transfected with 30–100 nM of miRIDIAN microRNA Mimics (Thermo Scietific –Dharmacon Lafayete CO) or 30–100 nM irrelevant mimic control using transfection reagent “Dharmafect duo” following the manufacturer's instructions. The amount of mimic was optimized for each miRNA. The efficiency of transfection was tested using irrelevant labeled mimic and flow cytometry. More than 95% of the cells were transfected. Mimic-transfected cells and their controls were cultured 48–72 hours, and then harvested and subjected to qRT-PCR. The irrelevant negative control sequence is based on mature sequence of cel-miR-67,: UCACAACCUCCUAGAAAGAGUAGA (Accession Number: MIMAT0000039. Cel-miR-67 has been confirmed to have minimal sequence identity with human, mouse and rat miRNAs.

### Western blot analysis

α-TC6, transfected either with 50 nM mimic miR-200c, mimic miR-182, , mimic miR-125b or irrelevant control were lysed in SDS, Tris-HCL buffer (pH 6.8), and aliquots corresponding to 1.8 to 2.6 µg of protein (for cMaf nuclear proteins) were subjected to Western blot analysis. Membranes were incubated either with polyclonal rabbit anti- cMaf (Bethyl Lab. Inc Montgomery TX) or rabbit anti-Zfpm2 antibody (Novus Biologicals Littleton CO) followed by anti-rabbit secondary antibody diluted 1∶25000. Chemiluminescent detection (Amersham Biosciences, San Francisco, CA) was utilized to evaluate the results. In order to normalize the samples the same membranes were stripped and re-probed with antibody anti SP1 (Abcam) for cMaf Western blots or anti β-actin (Sigma-Aldrich, St Louis, MO) for Zfpm2

### Inhibition of miRNA activity in insulinoma beta cell line Min6

Min6 were cultured in six well plate in DMEM medium containing 1.2 mg/ml glucose and 10% FBS, 24 hrs before transfection. Cells were transfected using Dharmafect duo transfection reagent (Dharmacon-ThermoScientific) with 200 nM each miRIDIAN Hairpin Inhibitor-miR125b; 182; and 200c or with 600 nM irrelevant inhibitor (Dharmacon-Thermo Scientific). The sequence of irrelevant inhibitor was based on cel-miR-67, mature sequence: UCACAACCUCCUAGAAAGAGUAGA (Accession number: MIMAT0000039. Cel-miR-67 has been confirmed to have minimal sequence identity with human, mouse, and rat miRNAs and show no identifiable effects on tested miRNA function. After 48 hrs culture in 37°C with 5% CO2 the cells were collected, washed 2× with PBS and stored in RNAlater. The Q-PCR was performed using TaqMan assays (Applied Biosystems-Invitrogen LaJolla CA) as described previously.

### Bioinformatic analysis

The target prediction databases were downloaded from corresponding database sites: PicTar prediction from http://pictar.mdc-berlin.de/; TargetScan from http://www.targetscan.org/; and Sanger prediction from http://www.ebi.ac.uk/enright-srv/microcosm/cgi-bin/targets/v5/download.pl. A Practical Extraction and Report Language (PERL) program was developed for this study. The targets for each miRNA of interest were detected by the program according to the PicTar, TargetScan and Sanger predication databases, respectively. For Pictar prediction the picTarMiRNA4way was selected (conserved in four species, human, mouse, rat and dog). For TargetScan we used the conserved version (conserved among mammals).

### Statistical analysis

Analysis of the miRNA array data was performed with the Significant Analysis of Microarray (SAM) method, using a False Discovery Rate (FDR) = 0.5% [33]. We used one-class SAM to compare the microRNA expression in α-cells versus that in β-cells. For this purpose we calculated the paired (α ΔCt−β ΔCt) differences for each preparation. ΔCt = RNA Sample Ct minus endogenous RNA Ct.

## Results

### miRNA signature in human pancreatic endocrine cells

**Figure 1:**
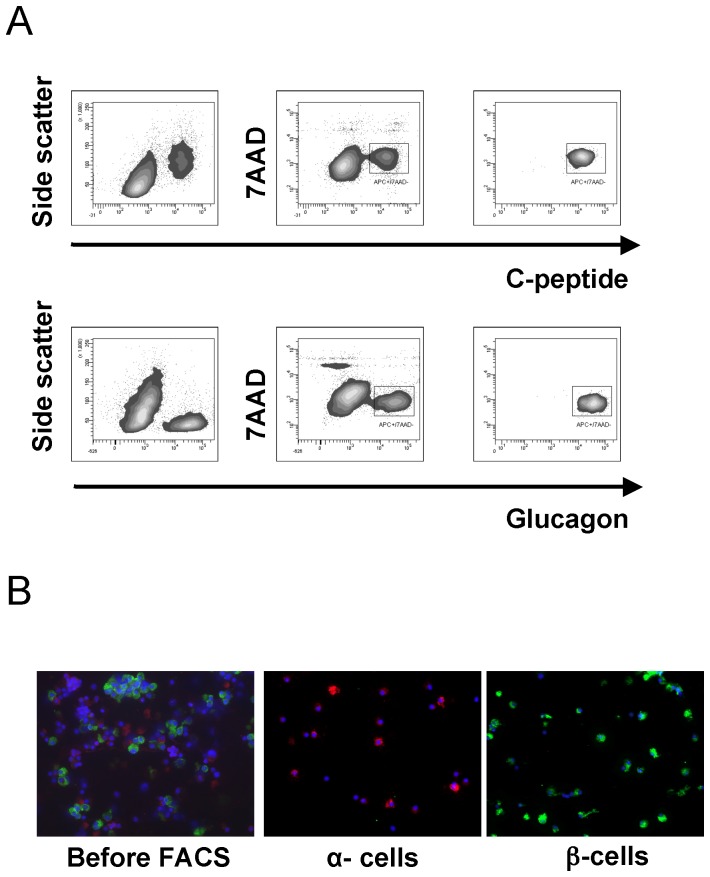
Intracellular staining and Flow-cytometric sorting of β-cells and α-cells. (A) c-peptide^+^/7AAD^−^ and glucagon^+^/7AAD^−^ dissociated human islet cells were isolated by FACS. The right panel shows the flow cytometric analysis of the isolated populations. (B) Immunostaining of islet cells before and after FACS. C-peptide positive cells are shown in green and glucagon expressing cells in red.

**Figure 2:**
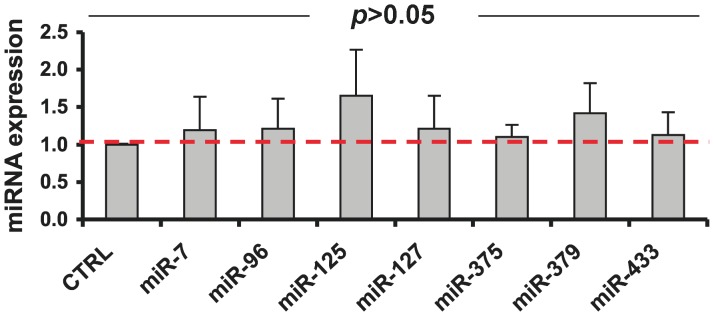
Fixation and permeabilization of human islets does not affect the content of miRNAs. Human islets were fixed and permeabilized as they were for FACS analysis. miRNAs expression was determined by qRT-PCR and fold changes (treated vs untreated islets) were expressed as mean +/− SD (n = 4). Control bar representing untreated islets is equal 1 also marked by the red dotted line. Paired, two-tail t test revealed no statistically significant differences in miRNAs expression (p>0.05).

In order to determine the differentially and commonly expressed miRNAs in sorted human α- and β-cells we compared their expression patterns. On average the purity of sorted α and β-cells was >98% (**Fig. 1** **and Table S2**). To confirm that the expression of miRNAs remained constant following the fixation and permeabilization of the cells, Q-RT-PCR comparing the expression levels in fixed and permeabilized cells versus untreated controls was performed with a panel of 7 primers highly expressed in islets [11], [12], [13]. The 7 primer panel comprised miR-7, miR-375, miR-96, miR-379, miR-433, miR-125b and miR-127. There were no significant differences in miRNA expression levels between the two groups (**Fig. 2**) indicating that the fixation method did not affect the yield of islet miRNAs.

The RNA samples from purified α- and β-cells were analyzed for miRNA expression using a PCR-based quantitative RT-PCR platform testing a total of 667 miRNAs. The analysis was performed in α- and β-cells isolated from 6 human pancreases. The cut-off for amplification was 32 cycles; only miRNAs detected in 4 out of the 6 samples were considered for analysis. We found 213 miRNAs fulfilling these conditions. Most of the miRNAs were more expressed in β-cells, with a fold change in expression of β- versus α-cells ranging from 1.6 to 112. 33 miRNAs were considered equally expressed in α- and β-cells with a fold change from 1.5 to 0.66 and 9 miRNAs were more expressed in α-cells with a fold change from1.5–2.9 as analyzed using the RNU48 small nucleolar RNA as endogenous control. Overall similar expression profiles were obtained using other small endogenous RNAs (**Table S3**). Despite using the same amount of RNA for each and every TLDA plate, we observed that in PFA-fixed tissue, the amount of endogenous RNAs is not proportional to total RNA. However, the differences were double sided. In some preparations the Cts were higher in β-cells while in others in α- cells (**Table S3**).

We performed a subsequent statistical analysis on this miRNA group to correct for expression of large numbers of genes applying the Significant Analysis of Microarray (SAM) [33]. SAM estimates the False Discovery Rate (FDR) by correcting the probability that genes were identified by chance. 141 miRNAs were identified as statistically significant, of which 134 were preferentially expressed in β-cells (β-miRNAs) and 7 in α-cells (α-miRNAs). The fold change for β-cells miRNAs ranged from 1.9 to 112 and for α-cells between 1.7 to 2.9 (**Table 1**).

**Table 1:** SAM analysis of miRNAs expressed in human α-and β-cells normalized with nucleolar RNU48 (shown in Table S3).

Upregulated	Score(d)	q(%)	FC	Upregulated	Score(d)	q(%)	FC	Upregulated	Score(d)	q(%)	FC
miR-204	6.41	0.00	108.42	miR-183	3.28	0.00	8.99	miR-30d	2.61	0.00	3.56
miR-432	5.51	0.00	106.15	miR-889	7.95	0.00	8.95	miR-7-1*	2.07	0.18	3.56
miR-370	7.14	0.00	88.65	miR-337-3p	2.59	0.00	8.72	miR-335	4.34	0.00	3.54
miR-433	7.21	0.00	81.27	miR-369-5p	2.44	0.00	8.52	miR-30a	3.85	0.00	3.54
miR-409-3p	3.88	0.00	76.52	let-7c	3.43	0.00	8.46	miR-132	2.00	0.18	3.49
miR-379	6.22	0.00	65.28	miR-190b	5.50	0.00	8.23	miR-200c	2.07	0.18	3.38
miR-127-3p	7.51	0.00	62.08	miR-485-3p	5.82	0.00	7.51	miR-30e*	3.49	0.00	3.37
miR-382	4.55	0.00	59.36	miR-376a*	3.01	0.00	6.86	miR-7-2*	2.24	0.00	3.36
miR-885-5p	5.95	0.00	57.50	miR-216b	2.32	0.00	6.74	miR-16	2.14	0.00	3.31
miR-136*	6.37	0.00	56.52	miR-29b-2*	3.17	0.00	6.74	miR-484	3.83	0.00	3.29
miR-148a	5.43	0.00	55.69	miR-154*	1.99	0.18	6.45	miR-101	1.98	0.18	3.26
miR-323-3p	7.35	0.00	54.66	miR-491-5p	4.91	0.00	6.32	miR-324-3p	3.02	0.00	3.09
miR-411	5.67	0.00	52.79	miR-27b	5.03	0.00	6.30	miR-29b	3.19	0.00	3.05
miR-183*	2.93	0.00	44.06	miR-134	2.57	0.00	6.27	miR-29a	3.27	0.00	3.01
miR-668	4.12	0.00	37.72	miR-654-3p	2.32	0.00	6.23	miR-671-3p	2.45	0.00	3.01
miR-30a*	4.44	0.00	31.66	miR-139-5p	2.26	0.00	6.09	miR-339-3p	2.02	0.18	2.98
miR-654-5p	2.21	0.00	31.30	miR-24	6.01	0.00	6.07	miR-301b	2.05	0.18	2.96
miR-337-5p	3.17	0.00	27.90	miR-380*	3.16	0.00	5.98	miR-320	3.21	0.00	2.95
miR-125b	5.45	0.00	27.70	miR-574-3p	4.54	0.00	5.95	miR-340	2.81	0.00	2.81
miR-494	7.65	0.00	23.45	miR-369-3p	3.09	0.00	5.88	miR-30e	2.69	0.00	2.78
miR-96	3.89	0.00	23.19	miR-130b	2.64	0.00	5.87	miR-340*	3.72	0.00	2.75
miR-539	5.29	0.00	22.25	miR-655	3.10	0.00	5.83	miR-186	2.23	0.00	2.74
miR-758	3.86	0.00	20.59	miR-22*	3.86	0.00	5.83	miR-7	2.52	0.00	2.73
miR-656	4.01	0.00	17.76	miR-29c	2.94	0.00	5.81	miR-103	2.71	0.00	2.71
miR-410	4.84	0.00	17.26	let-7b	3.69	0.00	5.36	miR-598	3.46	0.00	2.69
miR-376a	5.44	0.00	16.86	miR-324-5p	4.24	0.00	5.35	miR-99b	2.56	0.00	2.63
miR-15a	4.18	0.00	15.98	let-7a	2.41	0.00	5.34	miR-93*	2.37	0.00	2.62
miR-409-5p	4.07	0.00	15.32	miR-129-3p	4.30	0.00	5.34	miR-26a	3.05	0.00	2.56
miR-487b	4.57	0.00	15.14	miR-15a*	2.39	0.00	5.28	miR-145	2.95	0.00	2.48
miR-493*	2.96	0.00	14.49	miR-29c*	3.57	0.00	5.26	miR-92a	2.02	0.18	2.36
miR-493	2.78	0.00	13.49	miR-185	4.60	0.00	5.17	miR-374b	2.69	0.00	2.33
miR-22	3.31	0.00	12.69	miR-184	5.09	0.00	5.04	miR-429	2.36	0.00	2.26
miR-152	6.72	0.00	12.60	miR-21	5.68	0.00	4.90	miR-191	2.21	0.00	2.24
miR-376c	5.45	0.00	12.33	miR-331-3p	4.53	0.00	4.79	miR-744	2.08	0.18	2.23
miR-483-5p	3.42	0.00	12.12	miR-628-5p	2.74	0.00	4.71	miR-328	2.46	0.00	2.20
miR-543	5.14	0.00	12.11	miR-361-5p	2.01	0.18	4.58	miR-551b	2.45	0.00	2.20
miR-345	7.11	0.00	12.06	miR-200b*	2.32	0.00	4.36	miR-301a	2.35	0.00	2.13
miR-129-5p	3.11	0.00	11.82	miR-34a	2.11	0.00	4.20	miR-30c	2.97	0.00	2.09
miR-23b	6.10	0.00	11.70	miR-381	3.61	0.00	4.17	miR-195	1.91	0.18	2.09
miR-770-5p	2.96	0.00	11.62	miR-193b	4.17	0.00	4.02	miR-200a	1.93	0.18	2.07
miR-411*	2.46	0.00	10.89	miR-25	2.06	0.18	3.98	miR-26b	2.36	0.00	2.05
miR-495	5.89	0.00	10.84	miR-296-5p	2.44	0.00	3.93	miR-375	2.15	0.00	2.01
miR-182	3.27	0.00	9.80	miR-148b	2.86	0.00	3.77	miR-149	2.66	0.00	1.99
miR-154	3.00	0.00	9.30	miR-27a	3.94	0.00	3.67	miR-597	2.03	0.18	1.97
miR-99a	2.54	0.00	9.15	miR-203	3.41	0.00	3.62				

Downregulated	Score(d)	q(%)	FC
miR-221	−1.87	0.58	0.57
miR-194	−2.01	0.50	0.51
miR-181a	−2.07	0.50	0.51
miR-708	−2.27	0.34	0.47
miR-192	−2.12	0.50	0.47
miR-146a	−2.48	0.34	0.46
miR-192*	−2.90	0.26	0.34

The score (d) represents value of the T-statistic; a higher score means a greater difference between the two groups. Q-values correspond to the p-values adapted to the analysis of large number of genes. FC is the fold change of expression (β- vs α-cells).

### Bioinformatic analysis

MiRNAs regulate protein expression. Recent study showed that following miRNA overexpression, the major reason for reduced protein levels is destabilization of target mRNAs [34]. Therefore, to identify potential β-miRNA targets we compared our miRNA array data with previously published genome-wide mRNA (GWR) expression studies. We looked for potential targets of β-miRNAs in the BCGA –Beta Cell Gene Atlas- [35] using target RNA predictive algorithms. Only β-miRNAs with a fold change β vs α cells >3 were considered. The BCGA is a transcriptome atlas of pancreatic β-cell containing mostly genes identified by Massively Parallel Signature Sequencing (MPSS) analysis of human pancreatic islet samples and microarray analyses of purified rat α- and β-cells. The targets were selected by three of the most commonly used computational microRNA predictive target programs, namely miRBase, Targetscan and Pictar [36], [37], [38]. We analyzed 5 gene groups: genes with expression enriched in α-cells (αG), genes enriched in β-cells (βG), transcription factors expressed in both α- and β-cells (αβTF), and transcription factors specifically expressed in α-cells (αTF) or in β-cells (βTF) (**Table S4**).

### Overexpression of β-miRNAs in α-cells

cMaf [avian musculoaponeurotic fibrosarcoma (v-maf) oncogene homolog] and Zfpm2 (zinc finger protein, multitype 2-FOG2) are two transcription factors identified as exclusively expressed in α-cells [35] which contain multiple β-miRNA recognition sites in their 3′UTR (**Figure S1**). cMaf is a member of the large Maf family of transcription factors that is expressed in pancreatic islets [39]. cMaf is capable of regulating glucagon expression in α-cell lines [40], [41] through the specific G1 element of the glucagon promoter [41]. Zfpm2 (Fog2), a member of the Fog family of transcription factors, binds p85a, the pI3K regulatory subunit, inhibiting the formation of the IRS-1/p85a/p110 complex and subsequent activation of PI3K which results in attenuation of the insulin-signaling pathway [42].

**Figure 3:**
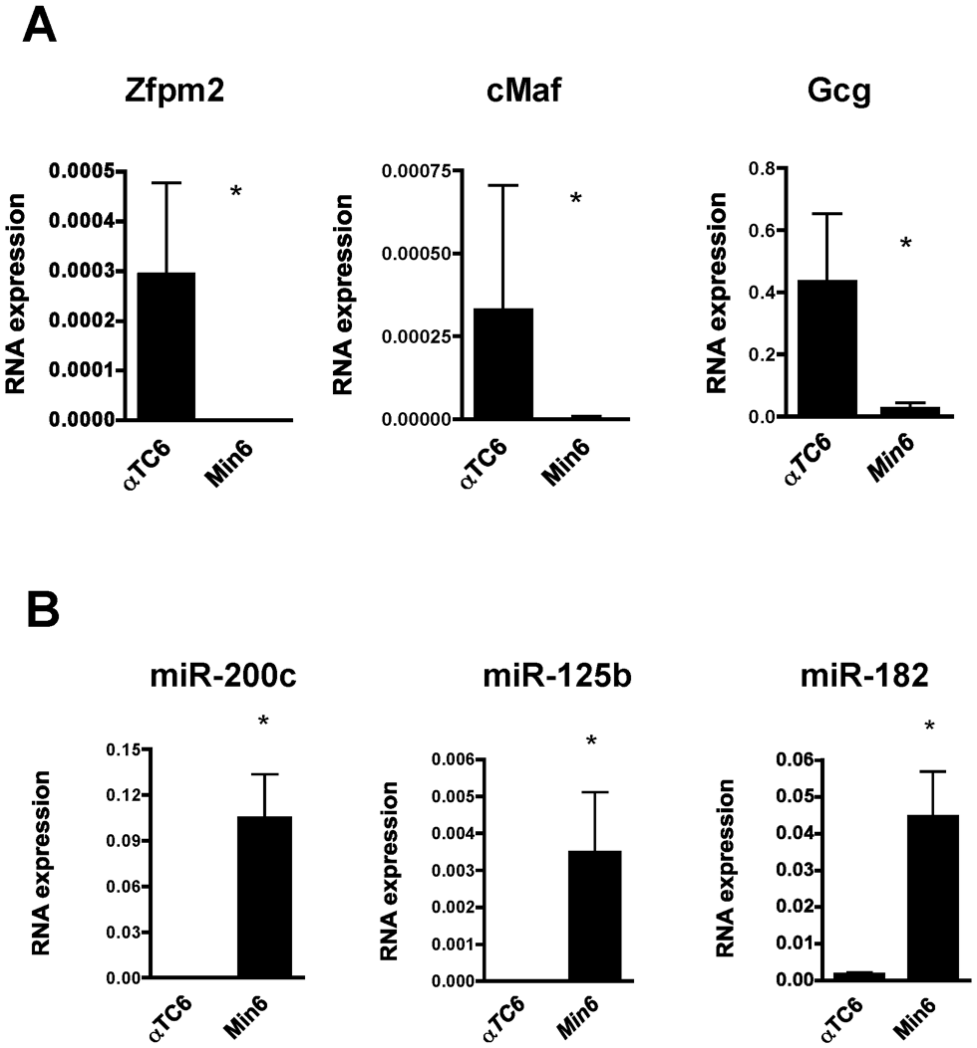
Expression of miRNAs and target RNAs in Min6 and α-TC6 cells. A. Differential expression of cMaf, Zfpm2 and Gcg mRNA assessed by qRT-PCR. Experiments are expressed as RQ = 2^−ΔCt^, ΔCt = (Ct_RNA gene_−Ct_RNA Ctrl_). Beta actin was used as endogenous control, mean ± SD (n = 4), * *p* = 0.04, 0.018 and 0.018 (t-test, 2 tails) for cMaf, Zfpm2 and Gcg respectively. **B.** Differential expression of β-miRNAs: miR-200c, miR-125b and miR-182 assessed by qRT-PCR. Experiments are expressed as RQ = 2^−ΔCt^, ΔCt = (Ct_RNA gene_−Ct_RNA Ctrl_). U6 small nuclear RNA was used as endogenous control, mean ± SD (n = 4), * *p* = 0.005, 0.005 and 0.0022 (t-test, 2 tails) for miR-200c, miR-125b and miR-182.

Among the β-miRNAs potentially targeting cMaf are miR-125b, miR-182 and miR-200c, which are 27.3-, 9.7- and 3.3 fold more expressed in β-cells (**Table 1**). In addition, miR-200c has been shown to target Zfpm2 [42]. Both miR-200c and Zfpm2 are conserved components of the insulin pathway, a critical regulator of metabolism and cell/tissue growth in animals. We first quantified expression of cMaf and Zfpm2 in glucagon-secreting αTC6 cells and insulin secreting Min6 cells, which were used as surrogates for α and β cells respectively. Compared to αTC6 cells, the expression of both genes, cMaf and Zfpm2, was very low in the β-cell line, which is in line with previous findings in human α- and β- cells [35] (**Fig. 3A**). Moreover, in Min6 the expression of miR-125b, miR-182 and miR-200c is much higher than in alpha TC6. In n = 4 independent experiments we found a 222 fold (Min6 vs alphaTC6) for miR-125b, 27 fold for miR-182 and 166×103 for miR-200c (**Fig. 3B**). The difference in expression is particularly high because miR-200c is almost undetectable in alphaTC6 cells. Judging by the results of these experiments we conclude that both cell lines can be used as surrogate for primary alpha and beta cells because the expression profile of both target genes and miRNAs is similar to primary cells.

**Figure 4:**
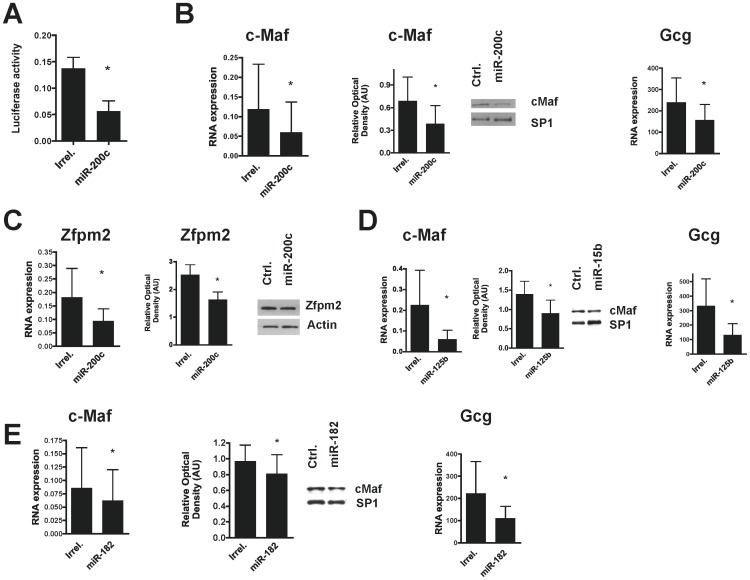
Over-expression of β-miRNAs in α-TC6 cells. A. miR-200c targets the 3′UTR of cMaf. Luciferase reporter assay performed in α-TC6 cells transfected with luciferase firefly reporter vector containing the 3′UTR of mouse cMaf and exogenous miR-200c or irrelevant negative control mimics. Luciferase activity was standardized by the Renilla luciferase, mean ± SD (n = 3), * *p* = 0.002 (t-test, 2 tails). **B.** miR-200c inhibits Maf, Gcg expression in α-TC6 cells. 72 hrs overe-xpression of mimic miR-200c (50 nM) inhibits the expression of endogenous cMaf (RNA and protein) and RNA Gcg, (n = 8), * *p* = 0.0078 and 0.0495 (Gcg), Wilcoxon signed rank test-2 tails. Densitometry analysis of cMaf western blots (n = 5) * *p* = 0.032 Wilcoxon signed rank test-2 tails. **C.** miR-200c inhibits Zfpm2 at RNA and protein levels, (n = 8), * *p* = 0.0078, Wilcoxon signed rank test-2 tails. Densitometry analysis of Zfpm2 western blots (n = 5), * *p* = 0.032. **D.** miR-125b inhibits c-Maf and Gcg expression in α-TC6 cells. Over-expression of miR-125b mimics (50 nM) for 72 hs inhibits endogenous cMaf and Gcg mRNAs, (n = 5), * *p* = 0.05 and 0.002 respectively, paired-2 tailed t test. Densitometry analysis of cMaf western blots (n = 5), *p =  0.001, paired -2 tailed t test. **E.** miR-182 inhibits cMaf and Gcg expression in α-TC6 cells. Over expression of miR-182 (50 nM) for 72 hs inhibits endogenous cMaf and Gcg mRNA, (n = 6), * *p* = 0.036 for cMaf and Gcg, Wilcoxon signed rank test-2 tails. Densitometry analysis of cMaf Western blots (n = 3), * *p* = 0.029. RNA assessment experiments are expressed as RQ = 2^−ΔCt^, ΔCt = (Ct_RNA gene_−Ct_RNA Ctrl_). Beta actin was used as endogenous control. Results are shown as mean ± SD. For densitometry calculations representative Western blots shown.

To demonstrate if the cMaf 3′UTR is a target of miR-200c, αTC6 cells were co-transfected with luciferase reporter containing the 3′-UTR of murine cMaf and exogenous mimic miR-200c or irrelevant negative control mimic. Compared to irrelevant negative control the transfected mimic miR-200c significantly down-regulated the 3′UTR reporter activity (**Fig. 4A**), indicating that miR-200c recognized the specific site on 3′-UTR of cMaf and could modulate the expression and function of this gene.

We then over-expressed miR-200c in αTC6 cells and analyzed the effects on cMaf and Zfpm2 expression. miR-200c down-regulated the expression of cMaf mRNA and protein. xpression of cMaf mRNA and protein. As expected, the lower levels of cMaf resulted in decreased expression of Gcg mRNA [40], [41] (**Fig. 4B**). The overexpression of miR-200c also downregulated Zfpm2 mRNA and protein levels (**Fig. 4C**). The same experiments were performed with miR-125b (**Fig. 4D**) and miR-182 (**Fig. 4E**) resulting in down regulation of endogenous cMaf and Gcg mRNAs as well cMaf protein levels. This result strongly suggests that miR-125b and miR-182 regulate cMaf as well.

**Figure 5:**
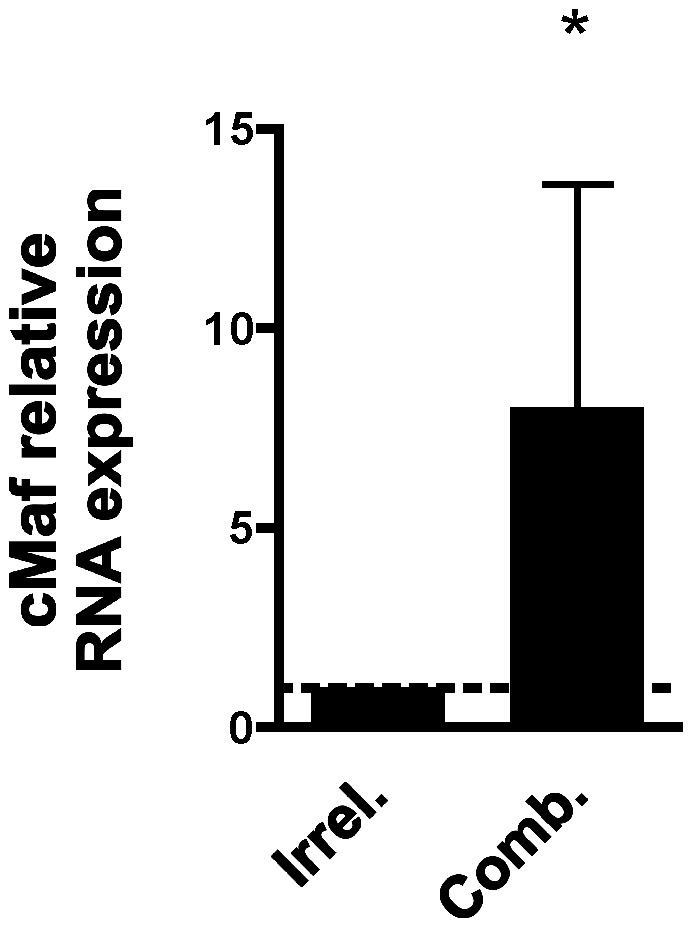
Inhibition of β-miRNAs in beta cells. Min 6 cells were transfected with hairpin inhibitors of miR125b, 182 and 200c (200 nM each) or irrelevant hairpin inhibitor control (600 nM). The cells were cultured for 48 hrs, collected and subjected to qRT-PCR. cMaf expression is shown as the fold change of miRNAs inhibitors over control treated samples calculated as RQ = 2^−ΔCt^, ΔCt = (Ct_RNA gene_−Ct_RNA Ctrl_). Beta actin was used as endogenous control, n = 6, *p = 0.029, Wilcoxon signed rank test-2 tails. Dotted line marks the relative control set to 1.

To test if the expression of miR-200c, miR-125b and miR-182 could contribute to the low expression of cMaf in beta cells, Min6 cells were transfected with a combination of 200 nM each miR-200c, miR-125b, and miR-182 exogenous hairpin inhibitors (Dharmacon-Thermo Scientific). miRNA inhibitors-treated cells showed statistically significant increase in the cMaf transcripts compared to cells treated with irrelevant control inhibitor (**Fig. 5**).

## Discussion

We determined the differential expression of miRNAs in human pancreatic α- and β-cells. For that purpose we sorted dissociated human islet cells utilizing glucagon and c-peptide as markers. This method has been used previously for sorting of mouse β-cells [25].

The SAM identified 141 miRNAs, of which 134 were expressed in β-cells with a relatively high fold increase of 1.9–112 (β-miRNAs) and only 7 miRNAs with moderate fold increase of 1.9–2.9 in α-cells (α-miRNAs).

Some β-miRNAs were previously associated with the regulation of insulin expression and content. For example, miR-204 is primarily expressed in insulinomas and co-localizes mainly with insulin [43]; miR-127-3p and miR-184 are positively correlated with insulin biosynthesis and negatively correlated with glucose-stimulated insulin secretion (GSIS) [44]; miR-148 controls the insulin content in β-cells through regulation of the insulin repressor SOX6 [45] and miR-29 contributes to pancreatic β-cell dysfunction in prediabetic NOD Mice [46], and affects the release of insulin from β-cells by silencing of monocarboxylate transporter (MCT1) [47]. MiR-375 and miR-7, known as islet miRNAs [11], [13], [48], [49], are only 2 and 2.7 fold more expressed in β-cells (**Table 1**). Assuming that the abundance of miRNAs is an indicator of their biological function, our results suggest that the suppressive action of miRNAs in β-cells is significantly higher than in α-cells.

It is accepted that the expression of a specific subset of miRNAs may have a crucial effect on the acquisition and maintenance of a given phenotype. Indeed it has been reported that miR-24, miR-26 and miR-148 contribute to characterization of β-cell identity and maintenance of β-cell phenotype by suppressing two known insulin transcription repressors, Sox6 and Bhlhe22 [45].

Our results underscore the recent study and emphasize the importance of miRNAs interaction with TFs [50], [51] which determines the cell phenotype and suggests that β-miRNAs could influence β-cell phenotype by restricting expression of transcription factors that determine the alpha cell phenotype. As a matter of fact, the over-expression of miR-200c down-regulates both cMaf mRNA and protein expression with the subsequent decrease in Gcg RNA and suppresses Zfpm2 at both RNA and protein levels, effectively attenuating the α-cell phenotype. Conversely, inhibition of miR-200c, miR-125b and miR-182 in Min6 cells increased the amount of cMaf transcripts. Interestingly, cMaf has been identified as a beta cell disallowed gene [52]. Disallowed beta genes are a group of genes whose expression is specifically inhibited in beta-cells to avoid the interference with critical beta cell functions. The presence of multiple β-miRNAs recognition sites in the 3′UTR of cMaf suggests that β-miRNAs contribute to this function (**Fig S1**). This is an observation that certainly warrants additional research. Other miRNAs have been proposed as regulators of beta cell disallowed genes such as miR-29 regulating MCT1, another disallowed beta cell gene [47], This is reminiscent of the observation by Stark et al. that miRNAs tend to target genes expressed in neighboring tissues [53]. The authors argue that this phenomenon, to which they refer as mutual exclusion, could be engaged to prevent expression of redundant mRNAs in developmental programs influencing cell lineage.

In addition to miRNas examined in this study, we identified many others that are differentially expressed in human pancreatic α- and β-cells and found their potential targets using bioinformatics. Our observations set the stage for further studies to specifically test the role of miRNAs and their candidate target molecules.

## Supporting Information

Figure S1 TargetScan analysis of β-miRNAs 3′UTR recognition sites in c-Maf and Zfpm2 mRNAs.
(TIFF)

Table S1 RNA yield and purity of sorted human α- and β-cells.
(XLS)

Table S2 Donor information, purity and yield of human α- and β-cells after FACS sorting.
(XLS)

Table S3 Expression of miRNAs in α- and β-cells. Global expression of miRNAs in α-and β-cells. Only miRNAs detected at less than 32 cycles in 4 out of the 6 analyzed samples were included. FC is the fold change in expression (β- vs α-cells) using all RNA endogenous controls included in the TLDA platform.
(XLS)

Table S4 Identification of potential β-miRNA targets by analysis of islet gene populations with TargetScan, Pictar and MIRANDA algorithms. The analysis was performed on β-miRNAs, FC (β- vs α)>3 normalized to nucleolar RNU48.
(XLS)
